# Quantifying morbidities by Adjusted Clinical Group system for a Taiwan population: A nationwide analysis

**DOI:** 10.1186/1472-6963-8-153

**Published:** 2008-07-21

**Authors:** Wui-Chiang Lee

**Affiliations:** 1 Department of Medical Affairs and Planning, Taipei Veterans General Hospital, Taipei City, Taiwan; 2 Institute of Hospital and Health Care Administration, National Yang-Ming University School of Medicine, Taipei City, Taiwan

## Abstract

**Background:**

The Adjusted Clinical Group (ACG) system has been used in measuring an individual's and a population's morbidities. Although all required inputs for running the ACG system are readily available, patients' morbidities and their associations to health care utilizations have been rarely studied in Taiwan. Therefore, the objective of this study was using the ACG system to quantify morbidities for Taiwanese population and to examine their relationship to ambulatory utilizations and costs.

**Methods:**

This secondary analysis examined claims data for ambulatory services provided to 2.71 million representative Taiwanese in 2002 and 2003. People were grouped by the ACG system according to age, gender, and all ambulatory diagnosis codes in a given year. The software collapses the full set of ACGs into six morbidity categories (Non-users, Healthy, Low-morbidity, Moderate-, High- and Very-high) termed Resource Utilization Bands (RUBs). Each ACG was assigned a relative weight (RW), which was calculated as the ratio of mean ambulatory cost for each ACG to that for the overall. The distribution of morbidities was compared between years 2002 and 2003. The consistency of the distributions of visits, costs, and RWs of each ACG were examined for a two-year period. The relationship between people's morbidities and their ambulatory utilizations and costs was assessed.

**Results:**

Ninety-eight percent of the subjects were correctly assigned to ACGs. Except for non-users (7.9 ~ 8.3%), most subjects were assigned to ACGs of acute and minor diseases and ACGs of moderate-to-high-morbid chronic diseases. The distributions of ACG-based morbidities were highly consistent (r = 0.949, *p < 0.001*) between 2002 and 2003. The ACG-specific visits (r = 0.955, *p < 0.001*), costs (r = 0.966, *p < 0.001*) and RWs (r = 0.991, *p < 0.001*) were correlated across two years. People grouped to the high-morbid ACGs had more visits and costs than those grouped to the low-morbid ACGs. Forty-six percent of the total ambulatory costs were spent by eighteen percent of the population, who were grouped to the High- and Very-high-morbidity RUBs.

**Conclusion:**

This study demonstrated the feasibility, validity, and reliability of using the ACG system to measure morbidities in a Taiwan population and to explain their associations with ambulatory utilizations and costs for the whole country.

## Background

Taiwan launched its single-payer National Heath Insurance (NHI) program in 1995. The NHI enrollees receive universal and comprehensive benefit coverage in ambulatory and hospitalized care. The ambulatory care includes primary care, dental care, preventive care, laboratory tests, diagnostic imaging, and prescription drugs [1]. The ambulatory care system of Taiwan consisted of 8,560 clinics and 540 hospital-based outpatient departments in 2003 [2]. Around 97% of clinics and 86% of the hospitals were privately owned; the remains were government-run [2]. In contrast with the primary care systems in many European and North American countries, Taiwan has no list or gate-keeping system. The NHI offers the insured complete freedom of choice among providers. Therefore, patients can seek ambulatory care at any clinic or hospital outpatient department, regardless of the severity of their illnesses [1].

The volume of ambulatory visits was very high after implementing the NHI, with a mean number of annual visits 14.4 per person [2]. The growing rate in total ambulatory cost also exceeded that in total hospitalized care. For the purpose of cost containment, the NHI completed its phase-in program of comprehensive global budgeting for the entire health system in 2002 [3]. The entire budget was divided to four sub-budgets: hospitals and their outpatients (65.8%), clinics (21.9%), dental care (7.9%), and Chinese medicine (4.4%) in 2003. Because 45% of the hospital budget was allocated for outpatient services, totally 60% of the budget was used for ambulatory care, not including those for Chinese medicine [2]. This figure has aroused public concern about whether ambulatory expenditures are used in alignment with people's actual medical needs.

Recent studies found that an individual's medical needs are correlated with his/her total morbidities rather than his/her particular disease [4-6]. For this reason, accurate methods are needed to estimate the morbidity burdens of specific individuals and populations; otherwise, the payment may become misaligned with medical needs [7]. In the 1990s, the Johns Hopkins Adjusted Clinical Groups (ACGs) system was developed to cluster morbidities into clinically meaningful categories of medical need based on the mix of diseases that treated by all providers over a defined time interval, typically one year [8-10]. The system uses all diagnostic codes from claims data to quantify morbidities for individuals and, when aggregates, for overall population [9,10]. The validity and reliability of the ACG system has been documented in the United States [9-11], Canada [7,12], Sweden [13-15], and Spain [16-18]. Nevertheless, the scales of these studies were often restricted by the health care systems or the comprehensiveness of data. So far, no ACG analysis has been conducted for a whole country.

All ambulatory cares in Taiwan are claimed and uploaded electronically to the NHI's data warehouse on a monthly basis. Theoretically, the Taiwan NHI should be the ideal setting for adopting the ACG system for the whole country because all required inputs for running the system are readily available. Although the Department of Health (DOH), Taiwan, has used the data to estimate disease prevalence rates [2], no study has measured morbidities for the entire population yet. Moreover, the association between population's morbidities and their utilizations has been rarely discussed [19-21]. By taking advantage of the comprehensive claims data, the objective of this study was using the ACG system to quantify morbidities for Taiwanese population and to examine their relationship to ambulatory utilizations and costs.

## Methods

### Study design and data retrieval

This retrospective study analyzed secondary data of Taiwanese population continuously enrolled in the NHI system between January 1, 2002 and December 31, 2003. The data set was issued by the Taiwan National Health Research Institute (NHRI) for research purposes. The database provides complete individual-level data for age, gender, diagnosis codes (ICD-9-CM), and expenditure for each ambulatory claim. In addition to primary care, preventive cares, neonatal and child vaccination shots, prenatal screenings, laboratory and radiological studies were all included as long as they were claimed with definite or provisional diagnoses. For the sake of privacy and confidentiality, all personal identifiers were encrypted before releasing the data. The application of the data sets was reviewed and approved by the NHRI.

The original databases consisted of 320 million claims for 22.6 million NHI enrollees. Because the volume of data exceeded the capability of the software, NHI enrollees were randomly sampled by selecting those whose birth date ended with "0" (*e.g.*, 10, 20 or 30). An individual-specific analytic file was constructed by retrieving and aggregating data for age, gender, all diagnosis codes, visits and expenditures reimbursed by the NHI program during the two 12-month periods in 2002 and 2003, respectively.

### ADG and ACG assignment

The ACG software (version 7.0) was used without modifying its grouping algorithm [22]. This algorithm enables classification of each diagnosis into one of thirty-two clinically cogent morbidity clusters, called Aggregated Diagnosis Groups (ADGs) according to the likely persistence of medical conditions, grade of severity, etiology, diagnostic certainty, and need for specialty care [22]. For each individual, the total number of unique ADGs, age and gender were used to group each case into one mutually-exclusive morbidity cluster, named ACG.

### ACG-specific visits, ambulatory costs and relative weights

Annual ambulatory cost was defined as the sum of the total ambulatory utilizations reimbursed by the NHI and the copayment paid by the individual for a given year. Mean ambulatory cost for each ACG was calculated by summing up the costs for the entire population assigned to a given ACG and then dividing that amount by the number of people in that ACG. People whose costs exceeded the 99^th ^percentile within each ACG were truncated to minimize the influence of outliers on mean cost and to parallel previous validation studies [7,23]. The same method was used to calculate the mean number of visits for each ACG in 2002 and 2003. All visits and costs for preventive care were included and analyzed in this study.

Each ACG was assigned a relative weight (RW), which was calculated as the ratio of mean ambulatory cost for each ACG to the mean ambulatory cost for the entire population. The ACG-specific RW was defined as a proxy parameter of relative resource use of the given ACG to the population mean. The RWs for 2002 and 2003 were calculated separately using the data for each year. A person who was assigned to an ACG with higher RW was regarded as having relatively higher morbidity burdens and more medical needs than the general population [9,10].

### Morbidity groups

The ACG system automatically collapsed the full set of ACG categories into six simplified morbidity categories (Non-users, Healthy-users, Low, Moderate, High, and Very-high) termed Resource Utilization Bands (RUBs). The ambulatory utilizations of each RUB were represented by the mean number of visits and mean cost of people assigned to that RUB. The mean morbidity burdens of each RUB were represented by the mean number of unique ADGs and mean RW of the people grouped in that RUB.

### Statistical analysis

The technical feasibility of running the ACG system on a Taiwan population was judged by the percentage of people who could be correctly assigned to ACGs. The quality of the claims data required for running the ACG system was evaluated by the percentage of non-grouped diagnosis codes [22]. Population morbidities were described and compared according to their assigned ADGs and ACGs between 2002 and 2003 using the Pearson's correlation method. Disease markers that were produced by the ACG system were compared to the disease registries released by the DOH [2]. Fifteen diseases that were prevalent in Taiwanese population and with the same working definition in both systems were selected for comparison (see Table 2). The association of ACG-specific visits, costs, and RWs between 2002 and 2003 was also examined by Pearson's correlation method. The relationships between the morbidities of a population and their ambulatory visits and costs were assessed. All costs were calculated in New Taiwan Dollars (NTD). The exchange rate for U.S. dollars (USD) to NTD was 34.5 in 2002 and 34.4 in 2003. All data were analyzed using STATA Version 8 (Stata Corp., College Park, TX, USA); *p *values were 2-sided, with the significance level set at 0.05.

## Results

### Morbidity patterns of Taiwanese population

Claims data for 2,709,124 NHI enrollees were retrieved for analysis (49.3% male; mean age 36.6 ± 20.1 years in 2002 and 36.9 ± 20.0 years in 2003). The quality of diagnostic coding was significantly better in 2003 than in 2002 according to the percentage of non-grouped diagnosed codes (1.6% vs. 2.0%, *p = 0.023*).

The study population had parallel ADG distributions between 2002 and 2003 (r = 0.99, *p < 0.001*) (Table 1). Thirty-four percent of the population had one to three unique ADGs, 46% had four to nine ADGs, and 10% had ten or more ADGs with an average of 5.0 unique ADGs per person in 2002 and 5.2 ADGs in 2003 (*p < 0.05*). The most frequently assigned ADG was "Time-limited: Minor-Primary infections" (ADG 2 – 79%) followed by "ADG 1: Time limited: minor" (39.8%), "ADG 34: Dental" (36.5%), "ADG 7: Likely to recur: discrete" (35.4%), "ADG 10: Chronic medical: stable" (32.3%), and "ADG 26: Signs/Symptoms: minor" (31.6%).

**Table 1:** Taiwanese morbidity patterns by ADG in 2002 and 2003

ADG & Description	Year 2002		Year 2003	
		
	N	%	N	%
1 Time Limited: Minor	1,006,048	39.8	1,019,484	40.4
2 Time Limited: Minor-Primary Infections	1,992,226	78.9	1,968,976	78.0
3 Time Limited: Major	235,366	9.3	237,030	9.4
4 Time Limited: Major-Primary Infections	449,075	17.8	445,353	17.6
5 Allergies	302,123	12.0	315,368	12.5
6 Asthma	87,299	3.5	82,509	3.3
7 Likely to Recur: Discrete	895,045	35.4	933,523	37.0
8 Likely to Recur: Discrete-Infections	564,424	22.4	551,992	21.9
9 Likely to Recur: Progressive	60,111	2.4	61,405	2.4
10 Chronic Medical: Stable	815,730	32.3	859,344	34.1
11 Chronic Medical: Unstable	470,946	18.7	476,131	18.9
12 Chronic Specialty: Stable-Orthopedic	108,891	4.3	112,290	4.4
13 Chronic Specialty: Stable-Ear, Nose, Throat	40,914	1.6	43,052	1.7
14 Chronic Specialty: Stable-Eye	393,814	15.6	398,962	15.8
16 Chronic Specialty: Unstable-Orthopedic	65,792	2.6	64,459	2.6
17 Chronic Specialty: Unstable-Ear, Nose, Throat	31,632	1.3	32,406	1.3
18 Chronic Specialty: Unstable-Eye	91,887	3.6	99,621	3.9
20 Dermatologic	312,630	12.4	315,398	12.5
21 Injuries/Adverse Effects: Minor	514,994	20.4	590,592	23.4
22 Injuries/Adverse Effects: Major	397,795	15.8	393,998	15.6
23 Psychosocial: Time Limited, Minor	23,037	0.9	22,938	0.9
24 Psychosocial: Recurrent or Persistent, Stable	189,041	7.5	187,296	7.4
25 Psychosocial: Recurrent or Persistent, Unstable	35,821	1.4	36,799	1.5
26 Signs/Symptoms: Minor	799,092	31.6	854,014	33.8
27 Signs/Symptoms: Uncertain	689,129	27.3	714,919	28.3
28 Signs/Symptoms: Major	421,569	16.7	431,382	17.1
29 Discretionary	199,908	7.9	195,420	7.7
30 See and Reassure	76,531	3.0	81,357	3.2
31 Prevention/Administrative	454,848	18.0	479,046	19.0
32 Malignancy	40,496	1.6	40,945	1.6
33 Pregnancy	51,640	2.0	48,975	1.9
34 Dental	930,053	36.8	930,816	36.9

**Table 2:** Comparison of disease prevalence rates by ACG and Department of Health (DOH), Taiwan

Disease markers	Prevalence rate (per 1,000 NHI enrollees)	
	
	DOH	ACG
Asthma	34.4	33.6
Benign prostate hypertrophy	15.0	15.6
Cataract	28.7	29.8
Chronic obstructive pulmonary diseases	38.0	36.0
Cardiac arrhythmia	18.5	18.6
Cerebrovascular diseases	21.1	21.5
Diabetes	44.4	50.0
Hepatitis	49.2	54.0
Hypertension	91.7	110.2
Malignant tumors	15.0	16.1
Otitis media	31.5	30.1
Parkinson diseases	2.78	3.00
Pulmonary tuberculosis	5.8	7.5
Complications in pregnancy, childbirth, and puerperium	20.0	19.1
Urinary tract stones	15.1	18.1

Table 2 lists the prevalence rates of fifteen disease markers produced by the ACG system and recorded on the official disease registries. The prevalence rates of acute illnesses were very similar between two resources, such as cataract, arrhythmia, cerebrovascular diseases, otitis media, and Parkinson diseases. Higher estimates by the ACG system than the disease registries were noted in some chronic illnesses like diabetes, hypertension, tuberculosis, hepatitis, and urinary tract stones.

Ninety-eight percent of the population could be appropriately classified into eighty-two ACGs [see Additional file 1]. The percentage of non-users (*i.e.*, ACG 5200) was 8.3% in 2002 and 7.9% in 2003. The population distribution for ACGs was highly consistent between 2002 and 2003 (r = 0.949, *p < 0.001*), but people were not equally distributed among ACGs. Fifty percent of the population was assigned to eleven ACGs, and 67% of the population was assigned to twenty ACGs. Most assigned ACGs were divided between the following two morbidity groups: people with acute and minor diseases such as ACG 0300, 2400, 1800, 2100 and 3400, and those with moderate- to high-morbid chronic diseases such as ACG 4910, 4920 and 5050.

### ACG-specific visits, costs and RWs

The statistics of mean visit, cost, and RW of each ACG in 2002 and 2003 are listed in the Additional file 1. The ACG-specific visits (r = 0.955, *p < 0.001*), costs (r = 0.966, *p < 0.001*) and RWs (r = 0.991, *p < 0.001*) were highly consistent between two years. Ninety-two percent of the population had at least one ambulatory encounter with an average 15.3 ± 14.2 visits and NTD 11,488 ± 52,917 cost in 2002; and 14.9 ± 13.9 visits and NTD 12,017 ± 35,495 cost in 2003. The ACG 5070 had the highest RW (5.691 in 2002; 6.023 in 2003) while ACG 1600 had the lowest RW (0.065 in 2002; 0.046 in 2003).

### Cost related to morbidities

The mean number of visits and costs of the people assigned to a given ACG was associated with its morbidity burdens. People assigned to ACGs with chronic, recurrent, unstable medical conditions or more ADG combinations required more costly care than those with acute, stable conditions or fewer ADG combinations [see Additional file 1]. Table 3 lists the population distributions and ambulatory utilizations among RUBs. In 2002, thirty-four percent of the population was classified as Healthy-users and Low-morbidity RUBs and they used 18.4% of total visits and 11.6% of total costs. Conversely, eighteen percent of the population was grouped as High- and Very-high-morbidity RUBs, but they used 37.2% of total visits and 46.3% of total costs.

**Table 3:** Utilizations and costs among resource utilization bands (RUBs) in 2002 and 2003.

RUBs	Population distribution (%)	Average number of ADGs	Relative weights	Average visit (% of total visits)	Average cost in NTD (% of total costs)
	Year 2002				
	
Non-users	8.3	0	0	0	0
Healthy users	10.0	1.4	0.15	4.9 (3.5)	1,806 (1.7)
Low-morbidity	23.8	2.9	0.39	8.8 (14.9)	4,362 (9.9)
Moderate	39.6	5.5	1.00	15.8 (44.4)	11,197 (42.1)
High	14.2	8.9	1.98	25.9 (26.1)	22,134 (29.8)
Very-high	4.1	11.7	3.83	38.6 (11.1)	42,762 (16.5)

	Year 2003				
	
Non-users	7.9	0	0	0	0
Healthy users	8.0	1.4	0.13	4.0 (2.3)	1,490 (1.1)
Low-morbidity	25.3	2.8	0.35	7.8 (14.5)	4,049 (9.3)
Moderate	40.4	5.5	0.99	14.8 (44.4)	11,252 (41.5)
High	14.3	8.9	2.01	25.6 (27.1)	23,468 (30.7)
Very-high	4.1	11.7	3.99	38.6 (11.1)	46,575 (17.4)

## Discussion

This study demonstrated the feasibility of using the ACG case-mix adjustment system to quantify a large population's morbidities in Taiwan. The administrative barriers to running the system are low because that all required inputs are routinely collected. The coding quality is acceptable given that the percentage of non-grouped diagnoses codes was lower than the 5% standard [22]. Moreover, the reliability of the system on Taiwanese population is documented because that the distributions of ADGs, ACGs, and RWs were quite consistent across the two years studied. Finally, the system was validated by the finding that higher ambulatory costs are associated with the accumulation and severity of morbidity burdens. Although the validity and reliability of the system on Taiwanese has been verified in two small-scale studies [20,21], this study highlights that the ACG system works very well for large datasets.

In contrast with previous ACG studies, the findings of this study are more significant for the following reasons. First, the scale of this analysis was larger than that of other studies conducted in the United States [9,10], Canada [7,12], Sweden [13,14], and Spain [17,18]. Second, previous large-scale studies have often been limited to specific population [14,18,24], health care programs [9,10], or geographic areas [7,12]. This study quantified, for the first time, morbidities on a national basis. The findings of this study are representative and can be benchmark information for further studies and applications. Third, this study might be one of the first ACG analyses conducted in Asia, where the health care behavior, culture, delivery systems, and health insurance systems were different to the European and North American countries. The study findings underline the robustness of the ACG system across these barriers.

The observed ACG-based morbidities for Taiwanese revealed several notable characteristics. First, the mean number of unique ADGs per person (5.0 to 5.2) was higher than that reported in Canada [7], Spain [18], and the United States [9,22]. Second, Taiwanese population had higher incidence of cases in almost all ADG categories than people in Canada [7], Sweden [14], Spain [18], and the United States [22]. No evidence from this or previous studies can support or reject the hypothesis that Taiwanese are less healthy than people of the other countries. However, because the ACG system is mainly based on NHI claims, several influential factors to the high morbidity burdens should be considered. First of all, the NHI provides broad ambulatory coverage and all of them are grouped by the ACG system. For instance, newborns and children receive physical checkup and scheduled vaccinations at clinics or hospitals, and thus the number of visits for ACG0100 and ACG0200 are quite high. For patients with chronic illnesses, they need to refill prescription drugs at ambulatory settings every one to three months. Another concern is the NHI's low cost sharing policy and people's moral hazard. In 2003, the mean annual medical cost was USD 824 for Taiwanese, which was lower than the figure of many European countries such as France (USD 3,145), Germany (USD 3,183), Italy (USD 2,179), and United Kingdoms (USD 2,392) [2]. The average cost per visit was USD 23.8 in 2003 and patients paid about 8.89% of this regardless of their utilization rates [1,20]. The de facto absence of a referral system and low copayment deters people from using the resources cautiously. Finally, the NHI's payment schemes to doctors also increased the likelihoods of high consultation rates. There were 32,390 western medicine doctors in 2003, about 14.3 doctors per 10,000 people [2]. One-third of these doctors work as general practitioners at clinics and they are paid entirely on a fee-for-service basis. On the other hand, most of the specialists practicing in hospitals are paid on a salary basis plus volume-based bonus benefits. Therefore, there are financial incentives for doctors to increase service volumes for all kinds of ambulatory care [1].

The third finding was the disproportional concentration of ambulatory resources to the high-morbidity population. The top 4% of the NHI population grouped into the Very-high-morbidity RUB had nine times more visits and thirty times higher costs than people grouped into the Healthy-users RUB. These high-morbidity populations often had more than one chronic illness and multiple co-morbidities, which combined to produce a complex and challenging clinical dynamic [25]. This finding was consistent with recent reports by Starfield *et al. *that health care needs correlate with total morbidity burdens rather than particular diseases [5,6].

It is worth noting that the healthy users and low-morbidity population also used considerable ambulatory resources in Taiwan. This finding was consistent with the statistics released by the DOH that a high percentage of the ambulatory resources were used in the treatment of symptoms or minor illnesses [2]. This figure raises a significant concern that whether ambulatory resources are used in alignment with population's medical needs, especially after implementing the global budget payment scheme. Given the limited budget, the utilization and cost by the healthy-users and low-morbidity population will inevitably decrease the budget for the high-morbidity patients. Moreover, physician fee paid by the NHI is same for all ambulatory consultations, regardless of patient's morbidities. There are incentives for doctors to treat low-morbidity patients. Additionally, the cost for outpatient services is generally lower than that of the inpatient care. For hospitals run by caped revenue budgets, there are also financial incentives to increase outpatient services and decrease inpatient capacities. Therefore, the NHI program is potentially threatened by steering resources away from the high-morbidity population if a growing share of the budget is used for the low-morbidity population. In fact, the NHI has noticed this issue and adopted several strategies. For instance, the NHI adopted the upper volume threshold policy for clinics and outpatient services in January 2001 [1]. The physician fees for ambulatory care are discounted above the pre-set volume limit. Furthermore, many over-the-counter drugs have been no longer reimbursed since 2004. After long-term negotiation with the legislature and health care providers, the NHI has increased patient's cost sharing for hospital outpatient services since July 2006.

### Study limitations

Some limitations of this study should be mentioned. First, although the quality of coding and the non-grouped diagnosis rate in this study was within an acceptable range, the accuracy of diagnoses has not been systematically estimated. Therefore, the validity of the ACG system is still subject to coding quality. In fact, the ACG system is relatively resistant to the inaccuracy of diagnosis because the exact diagnostic code is not of prime interest in the ACG system [26]. The point is that it belongs to the right cluster of diagnoses in terms of ADGs, resulting in the expression of each patient's health status as a combination of different types of morbidity [14]. Inaccuracy regarding a patient's registered health condition might have occurred only if very different diagnoses are assigned to different types of morbidity [17]. Second, physicians may assign provisional diagnoses during initial visits. This is particularly significant for patients with diseases that required bacteriological, biochemical, or radiological studies when making a diagnosis. For instance, the estimated prevalence rates of diabetes, hepatitis, and pulmonary tuberculosis by ACG system were higher than the official statistics. Orueta *et al*. have used "episode of care" as the morbidity observation unit which could offer a longitudinal view of health problems and decrease the effects of provisional diagnoses [17]. Third, previous studies showed that explanatory abilities of the ACG-based morbidities were quite good for the same-year and next-year ambulatory visits and costs but not as good for inpatient costs in Taiwan [20,21]. Therefore, this analysis was designed mainly based on ambulatory claims and the findings could not be generalized to inpatient settings.

### Policy implications

The influence of morbidities on medical needs has seldom been addressed in Taiwan. Although the DOH continuously monitors the prevalence of diseases, epidemiological statistics focus more on illnesses rather than on morbidities. Given the association between morbidities and ambulatory utilization and cost, many health care insurance programs in the United States have adopted the ACG system to assess the case mix of enrollees, estimate morbidities, adjust capitation rates [27,28], identify high-cost patients [29], and predict resource utilization accordingly [30]. The ACG system has also been adopted in Canada and several European countries to improve the efficiency and quality of medical care [12-17].

Although the accuracy and quality of diagnostic coding needs continuous monitoring and improvement, this study suggests that the health care authorities, the NHI, and policy makers can take advantage of nationwide, comprehensive and readily available datasets to regularly measure an individual's and a population's morbidities in Taiwan. Together with the findings in previous studies [20,21], the morbidity information can be considered by policy makers when budgeting and allocating ambulatory resources. Further, the ACG system can help providers to identify the high-morbidity groups likely to cost more than the population mean. These high-morbidity populations merit organized ambulatory services such as case management programs to improve their health care quality and, at the same time, decrease costs.

## Conclusion

By routinely collecting administrative data, the ACG system provides a potentially useful measure of morbidities within a population. This study demonstrated the feasibility, validity, and reliability of using the ACG system to measure morbidities in a Taiwan population and to explain their associations with ambulatory utilizations and costs for the whole country.

## List of abbreviations

ACGs: Adjusted Clinical Groups; ADGs: Aggregated Diagnosis Groups; DOH: Department of Health; NTD: National Taiwan Dollar; NHRI: National Health Research Institute; NHI: National Health Insurance; RUBs: resource utilization bands; RWs: relative weights; SD: standardized deviation.

## Competing interests

The authors declare that they have no competing interests.

## Authors' contributions

WCL participated in the design of the study, performed the statistical analysis, and draft the final paper.

## Pre-publication history

The pre-publication history for this paper can be accessed here:



## Supplementary Material

Additional file 1 Taiwanese morbidity patterns by ACGs in 2002 and 2003. The descriptions and comparisons of percentage distributions, visits, costs, and relative weights (RWs) of Taiwanese morbidity patterns by adjusted clinical groups (ACGs) between 2002 and 2003. *(Note: the Appendix is submitted as an additional file).* Click here for file
