# Supplementary material for: Quantifying morbidities by Adjusted Clinical Group system for a Taiwan population: A nationwide analysis
Source: BMC Health Serv Res. 2008 Jul 21;8:153. doi: 10.1186/1472-6963-8-153 (PMC2492856; doi:10.1186/1472-6963-8-153)
Supplement: Additional file 1 — Taiwanese morbidity patterns by ACGs in 2002 and 2003. The descriptions and comparisons of percentage distributions, visits, costs, and relative weights (RWs) of Taiwanese morbidity patterns by adjusted clinical groups (ACGs) between 2002 and 2003. (Note: the Appendix is submitted as an additional file). [file 1472-6963-8-153-S1.doc]

**Additional file 1**

Taiwanese morbidity patterns by ACGs in 2002 and 2003

(continued.)

(continued)
